# Photosynthetic Acclimation of Larch to the Coupled Effects of Light Intensity and Water Deficit in Regions with Changing Water Availability

**DOI:** 10.3390/plants13141891

**Published:** 2024-07-09

**Authors:** Lu Jin, Xiaoqian Song, Yu Shi, Xin Guan, Huimin Tang, Haiyan Huang, Jiaqi Chen, Zhonghua Zhang, Zhonghua Tang

**Affiliations:** 1 College of Chemistry, Chemical Engineering and Resource Utilization, Northeast Forestry University, Harbin 150040, China; 2 Key Laboratory of Forest Plant Ecology, Northeast Forestry University, Ministry of Education, Harbin 150040, China; 3 Engineering Research Center of Forest Bio-Preparation, Northeast Forestry University, Ministry of Education, Harbin 150040, China; 4 Heilongjiang Provincial Key Laboratory of Ecological Utilization of Forestry-Based Active Substances, Harbin 150040, China

**Keywords:** boreal forest, *Larix gmelinii* (Rupr.) Kuzen, light intensity, water deficits, adaptation

## Abstract

The impact of frequent water deficits on dominant tree species in boreal forests has received increased attention, particularly towards addressing the global climate change scenarios. However, the impacts of coupled light intensity and water deficit in the regeneration and growth of *Larix gmelinii* seedlings, a dominant species in China’s boreal forests, are still unclear. We conducted a dual-factor controlled experiment with four light intensities (natural sunlight, 50% shading, 75% shading, and 90% shading) and three soil water conditions (80%, 60%, and 40% soil saturated water content). The results showed that the coupling of light and water has a significant effect on the growth and development of *Larix gmelinii* seedlings. In 40% of the saturated soil moisture content, net photosynthetic rate, transpiration rate, chlorophyll a, and total phenol—leaf were significantly lower than the same light conditions under 80% soil saturated water content. Under the coupling treatment of 60% soil saturated water content and 50% shading treatment, the plant height increment, net photosynthetic rate, stomatal conductance, transpiration rate, chlorophyll a, and phenolic compound content were significantly higher than those of other coupling treatments; however, more than 75% shading inhibited photosynthetic parameters, chlorophyll a, total flavonoid—leaf, and total flavonoid—branch. Our results have important implications for forest management practices; they provide a scientific reference for the early growth of *Larix gmelinii* seedlings under the coupling of light and water and promote the survival and growth of seedlings.

## 1. Introduction

Boreal forests, also known as taiga forests, account for approximately 25% of the world’s forested area, prevailing within a circumpolar forest belt. They are primarily characterized by the predominance of species such as spruce, fir, larch, pine, birch, and aspen [1]. They provide a wide range of ecosystem services, including approximately 45% of the world’s stock of growing timber, water regulation, soil and biodiversity conservation, and non-wood forest products [2]. Boreal forests also play a crucial role in global climate regulation and mitigation and store one-third of the global terrestrial carbon stock [3]. Therefore, conservation and sustainable management are extremely important for improving the lives of people worldwide. However, boreal forests are also particularly vulnerable to forest disturbance, forest management practices, and climate change [4,5]. Promoting forest resilience and ecosystem service functions has become the main focus of forest restoration and management [2].

The boreal forests in China are primarily concentrated in the Great Xing’an Mountains of northeast China, which are located in the southern part of the boreal forest belt and are strongly affected by global warming. The area has experienced more than half a century of high-severity timber harvesting and the forest fires of the 1980s [4]. The natural larch forests were destroyed, and large areas were dominated by poplar and birch trees. Previously highly diverse and heterogeneous boreal ecosystems have experienced ecological simplification and decreases in functionality. To achieve the goal of improving ecosystem multifunctionality and services, the Chinese government has launched an ambitious plan to eliminate activity on mountains and reforest them. Since the late 1970s, the Chinese government has implemented several forest-relegated ecological restoration projects to reverse forest loss and degradation [6]. Numerous studies have focused on forest management practices aimed at improving ecosystem multifunctionality [7,8]. Based on existing knowledge of succession, reducing human interference and accelerating the succession of birch forests to larch–broadleaved mixed forests are considered the main methods for improving ecosystem function.

Some physiological and ecological studies have revealed the ecological adaptability of larch, competition between coniferous and broad-leaved species, and ecological niche overlap [9,10,11]. The success of the transformation from birch forests to larch–broadleaved mixed forests mainly depends on seedling performance because seedling survival, establishment, and high biomass accumulation are the key bottlenecks in the life history dynamics of trees and strongly rely on both abiotic and biotic factors [12,13]. *Larix gmelinii* (Rupr.) Kuzen is the most important native dominant species in China’s boreal forests. It was considered a sun plant. Environments with greater light intensity are also more suitable for smaller *L. gmelinii* seedlings [14]. However, the poor seedling establishment and slow seedling growth compared with that of birch have resulted in the failure of this species to successfully compete for light in early growth stages [15]. As part of the ongoing global change, boreal forests are experiencing increasingly severe water stress, which causes biomass loss [16], but the magnitude of damage may be species-dependent [17,18]. It has been reported that the radial growth of *L. gmelinii* decreased significantly in the last 40 years in the Daxing’an Mountains in northeastern China, and that this trait was significantly negatively correlated with the intrinsic water use efficiency of this species. Larch seedlings are less sensitive to water deficit than birch seedlings, and larch seedlings have a conservative water use strategy compared with that of *Betula platyphylla* [11]. Nonetheless, the interactive effect of water availability and light intensity on larch seedlings remains unclear under climate change. Forest gaps created by tree thinning promote the growth of larch seedlings under the canopy.

The adaptations of *L. gmelinii* seedlings to light intensity under different water deficit conditions have not been described. To better guide tree thinning practices under ongoing global climate change conditions, we analyzed the photosynthesis and growth response of *L. gmelinii* seedlings under light intensity and water deficit conditions through controlled experimental conditions. We hypothesized that *L. gmelinii* faces more extreme water deficits due to the significant changes in evaporation and permafrost degradation caused by global warming. Our specific objectives were to (1) examine whether the combined effect of light and water deficit could impose biomass accumulation and photosynthesis activity; (2) determine the gas exchange parameters by which *L. gmelinii* seedlings adapt to water deficit under full light conditions; and (3) examine whether the phenolic compounds responded positively or negatively to coupled light intensity and water deficit.

## 2. Materials and Methods

### 2.1. Plant Materials and Treatments

**Figure 1:**
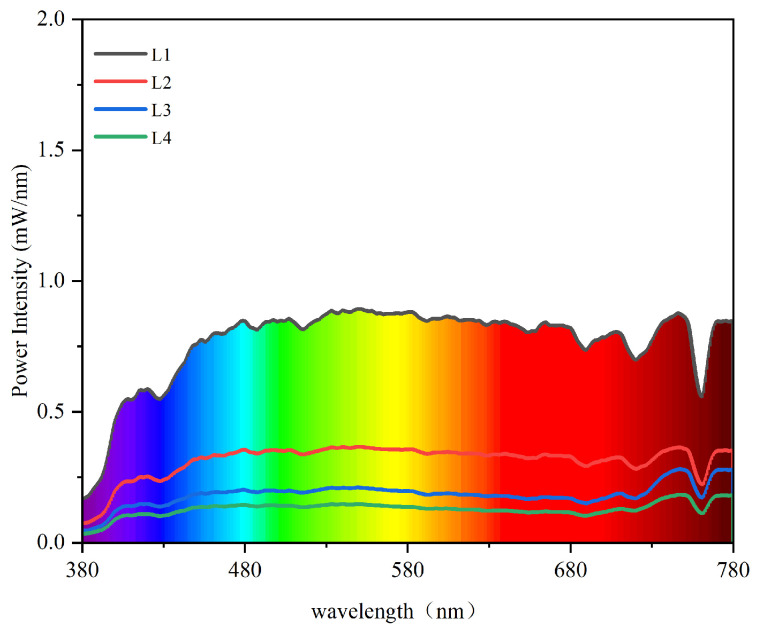
The spectrograms of different light intensities.

Two-year-old larch (*Larix gmelinii*) seedlings were collected from Mohe City, located in the core area of China’s boreal forests (50°11′–53°33′ N, 121°12′–127°00′ E) in April 2023. The average elevation of Mohe City is 313 m. The area belongs to the cold temperate continental monsoon climate. The annual average temperature is −5.5 °C, the minimum temperature is −53 °C, the maximum temperature is 38.3 °C, and the annual precipitation is 460.8 mm. The seedlings were potted in the botanical garden of Northeast Forestry University (45°43′15.99″ N, 126°38′25.05″ E). The mean annual temperature was 5.45 °C and the mean annual precipitation was 585 mm in 2000–2020 in Harbin. There are, on average, around 2570 sunshine hours per year. The plastic pots used for culturing the plants were filled with forest humus soil. After transplanting, the seedlings were allowed to regrow for two months under natural full light, and then the seedlings were divided into 4 light intensity gradients and 3 soil water content gradients, with a total of 12 groups (Table 1). Each group contained ten potted seedlings (Pot size: diameter × high × bottom diameter 16 × 17 × 12.5 cm, a seedling in each pot). To avoid experimental errors caused by different positions, the position of the pots was randomly moved every 7 days. The difference between light intensity gradients was measured by a spectrometer (SFIM-400; EVERFINE Corporation, Beijing, China) (Figure 1). The weighing method was used to control the soil water content of each treatment. Each pot was weighed at 4:00 pm every day to replenish the water. When it rains, at each shading level, the plants were covered with high density plastic, 150 microns thick, which provides top and side protection to prevent precipitation. Fine management was carried out during the experiment, and weeds were immediately removed upon discovery.

### 2.2. Measurement of Growth Response

The different coupling treatments of light intensity and soil water conditions were performed for 4 weeks. Plant height and basal diameter were measured before and after treatments. The biomass of leaf, stem, and root was harvested after 4 weeks of treatment. Plant height was determined by measuring the height from the stem base to stem tip, and the precision was 0.1 cm. A vernier caliper was used to measure the basal diameter, and the precision was 0.01 mm. After measuring the fresh weight of the leaf, stem, and root, plant materials were dried at 105 °C for 30 min and then dried at 80 °C to a constant weight to measure the corresponding dry weight.

### 2.3. Gas Exchange Measurements

A portable photosynthesis system 09 needle leaf chamber (Li-6400, Li-COR Inc., Lincoln, NE, USA) with a standard chamber was used to determine the net photosynthetic rate, stomatal conductance, intracellular CO_2_ concentration, and transpiration rate of the leaves from 9:00 to 11:00 on clear days. The second or third cluster of fully expanded leaves from the top was clamped into a 2 × 3 cm leaf chamber provided with a 1500 μmol·m^−2^·s^−1^ light intensity, which are realistic midday saturating light intensities for our location. Gas exchange measurements were performed with 9 repetitions from 9 different clusters of three plants.

### 2.4. Chlorophyll Contents Determination

To determine the chlorophyll contents, the needle leaves of three plants were collected and divided into three parts. A total of 0.025 g of needles per part was placed in glass tubes containing 5 mL of dimethyl sulfoxide for pigment extraction. All glass tubes were kept in the dark at 60 °C for 2 h, as described by Wang et al. [19]. The absorbance of each chlorophyll sample was measured at 480, 649, and 665 nm wavelengths using a spectrophotometer (723PC, Shanghai Jinghua Technology Co., Ltd., Shanghai, China). The chlorophyll a, chlorophyll b, and carotenoid contents were calculated by using equations provided by Wang et al. [19].

### 2.5. Chlorophyll Fluorescence Characteristics Analysis

The fluorescence characteristics of chlorophyll were determined using a pulse amplitude modulation fluorometer (PAM-2500, Walz, Effeltrich, Germany). The second or third cluster of fully expanded leaves from the top of each plant was collected from 3 different pots and pasted tightly to a flat surface to form leaf disks. Three leaf disks per treatment were acclimated to the dark for at least 30 min. The slow kinetic curves were generated according to previously described instructions. The minimal fluorescence (F0) under a radiation intensity of <0.2 μmol·m^−2^·s^−1^ photon was used to induce significant changes in fluorescence.

### 2.6. Determination of the Total Contents of Flavonoids and Phenols

The total flavonoid content was determined according to the nitrite method described by Wolfe et al. [20]. The plant sample (0.2 g) was accurately weighed and divided into three parts. Fifty milliliters of 60% ethanol was added to the tube, followed by centrifugation at 12,000 r·min^−1^ for 10 min in an 80 °C and a water bath for 30 min. The supernatant was diluted to 100 mL with 60% ethanol. Two milliliters of extract was added to a new test tube, 0.3 mL of 5% sodium nitrite was added, and the mixture was mixed well and allowed to stand for approximately 10 min. Then, 0.3 mL of 10% aluminum nitrate was added and the mixture was shaken well and allowed to stand for approximately 10 min. Finally, 4 mL of 4% NaOH was added, and the mixture was allowed to stand for 5–6 min. The absorbance at 510 nm was measured by an ultraviolet spectrophotometer (723PC, Shanghai Jinghua Technology Co., Ltd., Shanghai, China), and the total flavonoid content was calculated according to the following formula.

Total flavonoid content (mg/g) = (C × V)/M

where C is the total phenol concentration (μg/mL); V is the volume of the crude extract (mL); and M is the weight of the sample (g).

The total phenolic content was extracted and determined by the Folin–Ciocalteu method as described by Eduardo et al. [21]. Larch needles (0.2 g) were cut into three sections, which were subsequently divided into three parts. Then, 5 mL of 70% methanol was added to a 70 °C water bath for 10 min. After cooling at 12,000 r/min for 10 min, the supernatant was centrifuged at 70% methanol to a constant volume of 100 mL. Ten milliliters of the test solution was added, followed by five milliliters of 10% Folin phenol. The mixture was mixed well and then allowed to stand for 10 min. Then, 4 mL of 7.5% anhydrous sodium carbonate was added, and water was added to the mixture. An ultraviolet spectrophotometer (723PC, Shanghai Jinghua Technology Co., Ltd., Shanghai, China) was used for the analyses. The absorbance at 765 nm was determined, and the total phenol content was calculated.

Total phenol content (mg/g) = (C × V)/M

where C is the total phenol concentration (μg/mL); V is the volume of the crude extract (mL); and M is the weight of the sample (g).

### 2.7. LC-MS Analysis of Phenolic Compounds

We detected plant main defense substance (phenolic metabolism)-related compounds by referring to the method of Liu Yang et al. [22], which we adapted slightly: we used 0.2 g of dried powder of larch needles with good precision weight, and they were divided into three parts. We put them into a 5 mL centrifuge tube and added 3 mL 70% methanol. Ultrasonic treatment (100 kHz) was performed at 40 °C for 45 min. This process was repeated twice. After ultrasonication, the supernatant was extracted with a centrifuge at 12,000 r for 10 min. The supernatant extracted by two centrifugations was combined and evaporated using a rotary evaporator. After that, all the samples were redissolved with 1 mL of 70% methanol, centrifuged at 12,000 r for 15 min, and the supernatant was stored in a refrigerator at −20 °C for later use. Before the machine was centrifuged at 12,000 r again for 15 min, 800 μL of supernatant was taken and sent to the mass spectrometer.

The chromatographic separation system consists of ultra-high-performance liquid chromatography equipped with an LC-20AD pump, temperature controller, column temperature box, SIL20A automatic sampler (Waters, Shanghai, China), and BEHC18 chromatographic column (1.7 um, 2.1 mm × 50 mm) equipped with the same type of pre-column. The column temperature is used at 30 °C. The solvent system of 0.05% acetic acid–water (A) and 0.05% acetic acid–acetonitrile (B) was used for gradient elution at a flow rate of 0.25 mL·min^−1^: 5% B–95% B in 0–23 min; 95% B–5% B in 23–25 min; and 5% B in 25–31 min. The mass spectrometer was operated in positive ion mode in the range of 50–1000 *m*/*z*.

### 2.8. Statistical Analysis

The experimental data were organized with Microsoft Excel 2021. All the data were tested for normality using the Shapiro–Wilk test (*p* > 0.05). One-way analysis of variance (ANOVA, *p* < 0.05) was used to determine the significant differences among treatments, and then the least significant difference (LSD) test was performed. Two-way ANOVA was conducted using the IBM SPSS statistical package version 26 to explore the effects of light intensity and water deficit on *L. gmelinii*. The target compounds were identified by comparing their retention times to those of standard reference compounds. Peak values in different tissues and treatments were employed and normalized for further evaluation. The correlation analysis and figure development for each index were carried out by Origin 2022 and RStudio software ver 4.3.2.

## 3. Results and Analysis

### 3.1. Plant Growth Response to Coupled Light Intensity and Water Conditions

The growth parameters showed significant differences in light, water, and their coupling, and the effect of water on biomass was not significant (Table 2).

**Figure 2:**
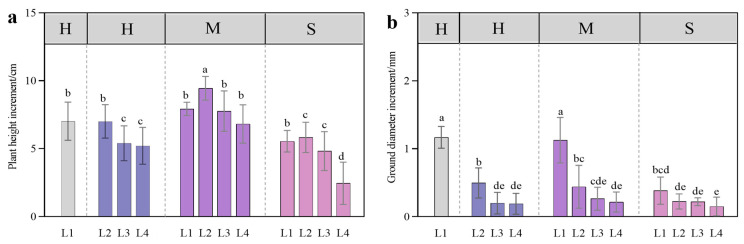
Plant height increase (**a**) and ground diameter increase (**b**) in *L. gmelinii* in response to light intensity and water conditions. All the data were statistically analyzed using one-way analysis of variance (ANOVA, *p* < 0.05) and the least significant difference (LSD) test. The data are presented as the means ± SDs (*n* = 10). Different lowercase letters indicate significant differences between treatments.

Throughout the experiment, the larch seedlings maintained normal growth, and the survival rate was 100%. At a certain soil water content, the plant height under the 50% shade (L2) treatment was greater than that under the other light treatments, and the ground diameter under the full light (L1) treatment was greater than that under the other treatments. The plant height was greatest under the L2-M treatment (Figure 2a), and the ground diameter was greatest under the L1-H treatment (Figure 2b). At 40% soil saturated water content, the plant height and the ground diameter under the different light treatments were lower than those under the 80% and 60% soil saturated water content treatments (H and M) (Figure 2a,b).

**Figure 3:**
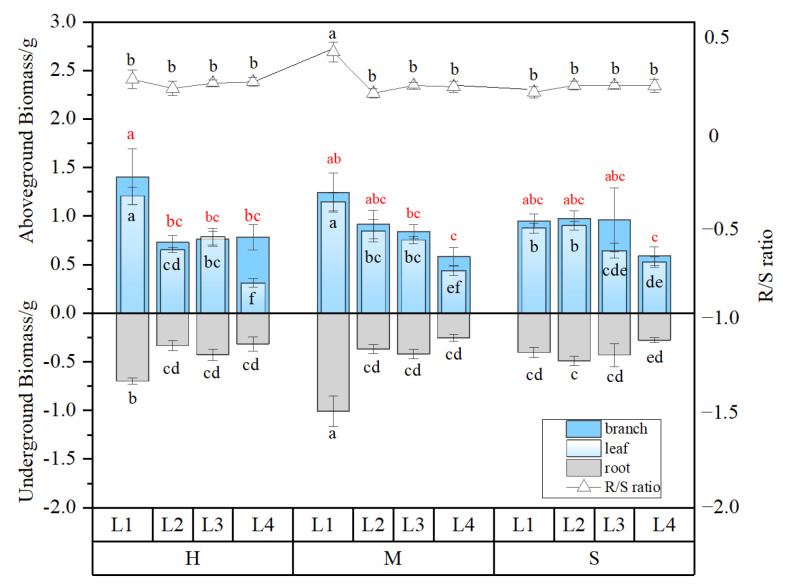
Biomass distribution of *L. gmelinii* seedlings under different water and light conditions. All the data were statistically analyzed using one-way analysis of variance (ANOVA, *p* < 0.05) and the least significant difference (LSD) test. The data are presented as the means ± SDs (*n* = 5). Different lowercase letters indicate significant differences among the different light and moisture treatment groups (*p* < 0.05).

Under H and M water conditions, the biomass of the roots and leaves was significantly higher than that of other light treatments under the same water condition under L1 light condition and under L4 light condition, and the biomass of the leaves was significantly lower than that of other light treatments under the same water content (*p* < 0.05). There was no significant difference in branch biomass under different light treatments under S water (*p* > 0.05). R/S under L1-M treatment was significantly higher than other treatments (Figure 3).

### 3.2. Gas Exchange Response to Coupled Light Intensity and Water Conditions

**Figure 4:**
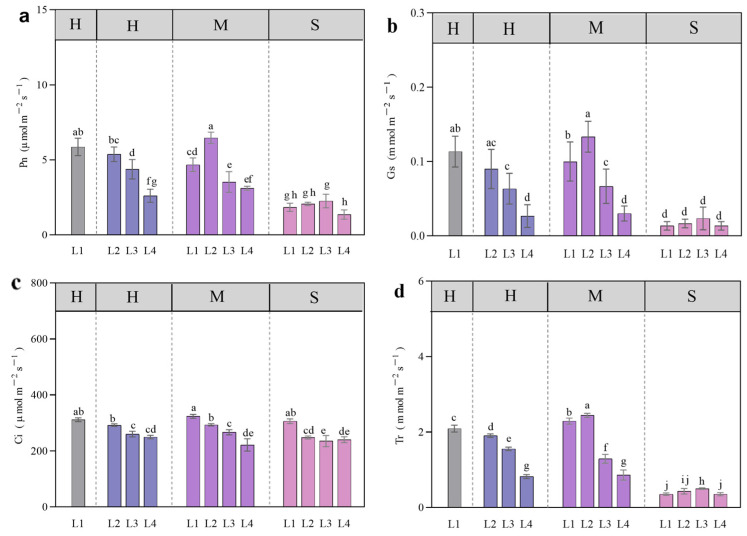
Gas exchange parameters of larch seedlings under different light and moisture treatments. Net photosynthetic rate (Pn) (**a**), stomatal conductance (Gs) (**b**), intercellular carbon dioxide (Ci) (**c**), and leaf transpiration rate (Tr) (**d**). All the data were statistically analyzed using one-way analysis of variance (ANOVA, *p* < 0.05) and the least significant difference (LSD) test. The data are presented as the means ± SDs (*n* = 3). Different lowercase letters indicate significant differences between treatments.

Under each water treatment (M, S), different light treatments showed that Ci under L1 light was significantly higher than that under other treatments (*p* < 0.05). Under H water, Ci under L1 and L2 treatments was significantly higher than that under L3 and L4 treatments (Figure 4c). Pn, Tr, and Gs under L1 and L2 conditions were significantly higher than those under L3 and L4 conditions under H water content (*p* < 0.05), and the L2 light treatment was significantly higher than those of L1, L3, and L4 light treatments under M water content (*p* < 0.05). Under S water, Pn, Tr, and Gs of different light treatments were significantly lower than those under H and M water treatments (*p* < 0.05) (Figure 4a,b,d).

**Figure 5:**
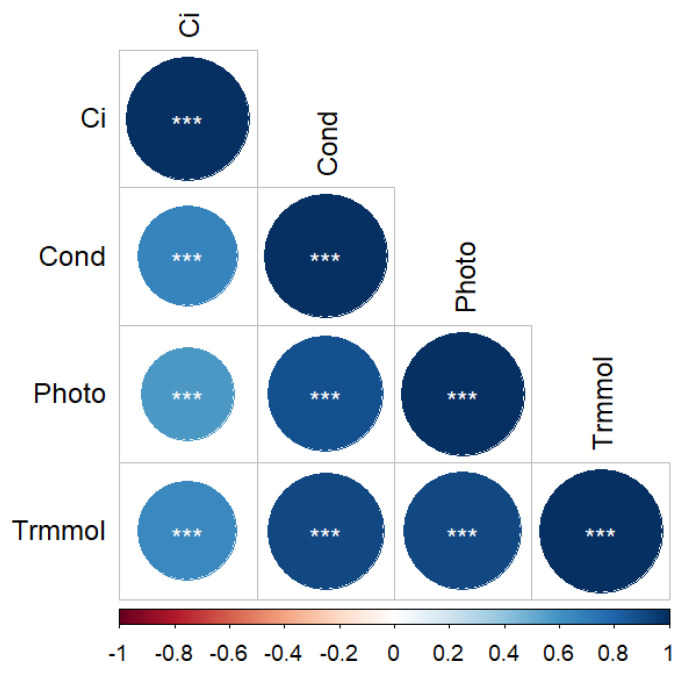
Correlation of photosynthetic parameters of *L. gmelinii* under different light and water treatments. The legend represents the level of correlation. *** in the graph indicate significant differences at *p* < 0.001.

Ci was significantly positively correlated with Pn, Tr, and Gs (*p* < 0.001), with values of 0.58, 0.65, and 0.66, respectively (Figure 5).

### 3.3. Changes in the Photosynthetic Pigment Content in Response to Coupled Light Intensity and Water Conditions

Light and water had significant effects on the chlorophyll content. The combination of light and water had significant effects on *Chla* and *Chla+b* but had no significant effects on *Chla/b* and *Chl/Car* (Table 2).

**Figure 6:**
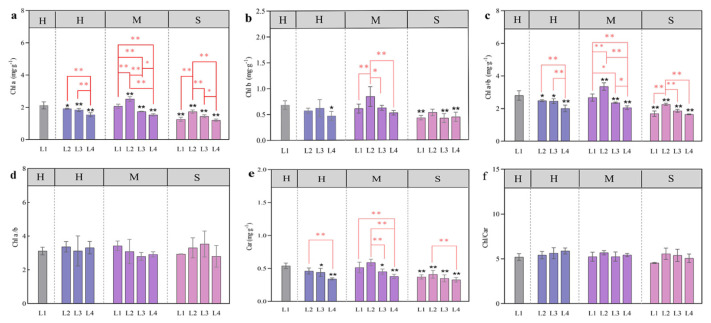
Effects of different light and water treatments on the photosynthetic pigment content of larch seedlings. *Chl a* (**a**), *Chl a* (**b**), *Chl a+b* (**c**), *Chl a/b* (**d**), *Car* (**e**), and *Chl/Car* (**f**). All the data were statistically analyzed using one-way analysis of variance (ANOVA, *p* < 0.05) and the least significant difference (LSD) test. The data are presented as the means ± SDs (*n* = 3). Black * (*p* < 0.05) and ** (*p* < 0.01) indicate significant differences between the control light intensity and water deficit treatments. For the same water treatment, significant differences among the different light level treatments are indicated by a red * (*p* < 0.05) and ** (*p* < 0.01) above the connecting line.

With the decrease in light intensity, *Chla, Chlb*, *Car*, and *Chla+b* showed a downward trend under H water treatment, and the L4 light treatment was significantly lower than other treatments (*p* < 0.05) (Figure 6a–d). With the decrease in light intensity, the contents of *Chla*, *Chlb*, *Car*, and *Chla+b* were the highest under L2 light conditions under M and S water conditions, and the L2-M treatment was significantly higher than other treatments (*p* < 0.05) (Figure 6a–d). There was no significant difference in *Chla/b* and *Chl/Car* under different light and water coupling treatments (Figure 6d,f).

### 3.4. Changes in Chlorophyll Fluorescence in Response to Coupled Light Intensity and Water Conditions

**Figure 7:**
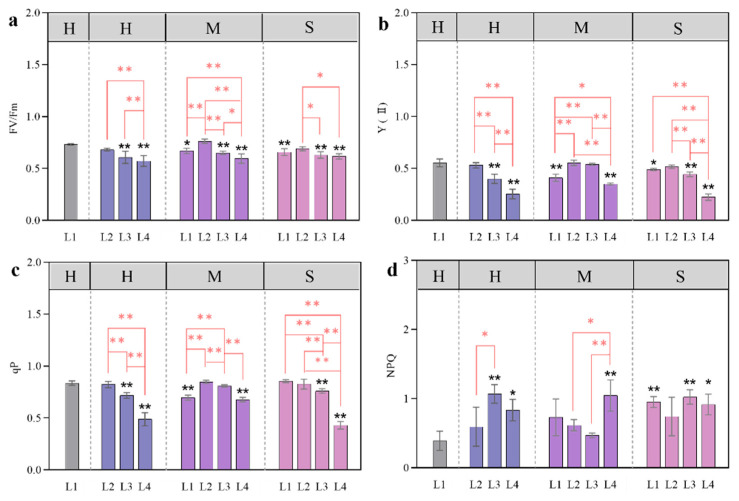
Effects of different light and water treatments on the chlorophyll fluorescence of larch seedlings. Fv/Fm (**a**), Y(II) (**b**), qP (**c**), NPQ (**d**). All the data were statistically analyzed using one-way analysis of variance (ANOVA, *p* < 0.05) and the least significant difference (LSD) test. All the data were statistically analyzed using one-way analysis of variance (ANOVA, *p* < 0.05) and the least significant difference (LSD) test. The data are presented as the means ± SDs (*n* = 3). Black * (*p* < 0.05) and ** (*p* < 0.01) indicate significant differences between the control light intensity and water deficit treatments. For the same water treatment, significant differences among the different light level treatments are indicated by a red * (*p* < 0.05) and ** (*p* < 0.01) above the connecting line.

Under certain soil water conditions, the changes in the chlorophyll fluorescence characteristics of larch seedlings under different light intensities varied, and the degree of change in some light treatments was significant. Fv/Fm, Y(II), and qP decreased gradually with decreasing light intensity under the H moisture treatment. Under the M and S moisture treatments, with the decrease in light intensity, there was an increase followed by a decrease (Figure 7a–c). With the decrease in light intensity and water content, NPQ showed an overall upwards trend, which was greater than that observed in the control (L1-H) (Figure 7d).

Under certain soil water conditions, the Fv/Fm, Y(II), and qP of L3 and L4 decreased. Compared with those of the control (L1-H), except for the L3-M of Y (II) and qP, the values in the other treatments decreased significantly (*p* < 0.05).

There was no significant difference in Fv/Fm, Y(II), or qP between the L2 and the control (L1-H) under the different water treatments. The L2 under the M treatment was the greatest, increasing by 4.23%, 0%, and 1.31% compared with those under the control treatment.

Compared with that of the control (L1-H), the NPQ of all the other treatments increased; the values in the L2 light treatment within each water treatment group were not significantly different from that of the control (*p* < 0.05). Under the H and S conditions, L3 and L4 increased significantly, by 63.30% and 53.09% and 61.75% and 57.2%, respectively. Under the M moisture treatment, L4 increased significantly, by 62.49%. PSII and qP were significantly lower in the L4 treatment group than in the other shading treatment groups, indicating that the photosynthetic apparatus of larch leaves may be partially inactivated or damaged under severe shading conditions.

### 3.5. Total Phenols and Total Flavonoids Associated with the Coupling of Light Intensity and Water Conditions

The coupling of light and water had a significant effect on total flavonoids, but only on total phenol—branches. Water treatment had a significant effect on total phenols, and light treatment had a significant effect on total flavonoids (Table 2).

**Figure 8:**
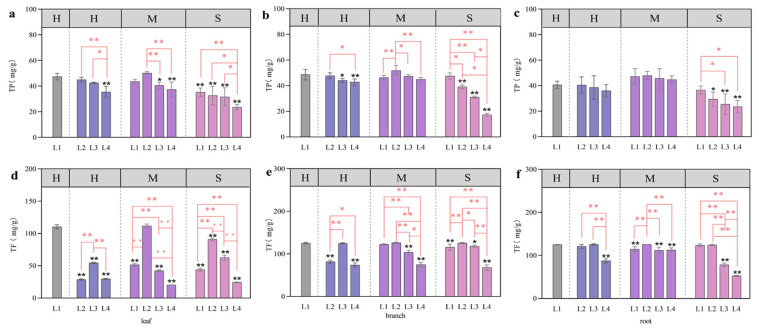
Effects of different light and water treatments on the total phenols and total flavonoids of larch. TP—leaf (**a**), TP—branch (**b**), TP—root (**c**), TF—leaf (**d**), TF—branch (**e**), and TF—root (**f**). All the data were statistically analyzed using one-way analysis of variance (ANOVA, *p* < 0.05) and the least significant difference (LSD) test. The data are presented as the means ± SDs (*n* = 3). Black * (*p* < 0.05) and ** (*p* < 0.01) indicate significant differences between the control light intensity and water deficit treatments. For the same water treatment, significant differences among the different light level treatments are indicated by a red * (*p* < 0.05) and ** (*p* < 0.01) above the connecting line.

The total phenolic contents in the leaves, branches, and roots first increased and then decreased with decreasing light intensity under the H and M treatments and decreased with decreasing light intensity under the S treatment. The content of each plant part was the highest under the L2-M treatment, but there was no difference from that under the L1-H treatment (*p* < 0.05) (Figure 8a–c). At a certain soil water content, the total flavonoid contents in leaves, branches, and roots first increased and then decreased with decreasing light intensity. The total flavonoid content in the leaves was 0.74% greater in the L2-M treatment group than in the control group (L1-H), but the difference was not significant. The content of total flavonoids in the branches under L1-H was the highest, followed by that under the L2-M treatment, but the difference between the two treatments was not significant (Figure 8d–f). Compared with that in the L1-H treatment, the total flavonoid contents in the roots of the L3-H, L2-M, and L2-S treatments were greater at each water content, with increases of 2.69%, 2.85%, and 2.43%, respectively; however, the differences were not significant (Figure 8f). The content of total flavonoids in the branches was lowest in the L2 and L4 treatments under the H water treatment and lowest in the L4 treatment under the M and S water treatments (Figure 8e).

### 3.6. Responses of the Phenolic Compound Content to Coupled Light Intensity and Water Conditions

**Figure 9:**
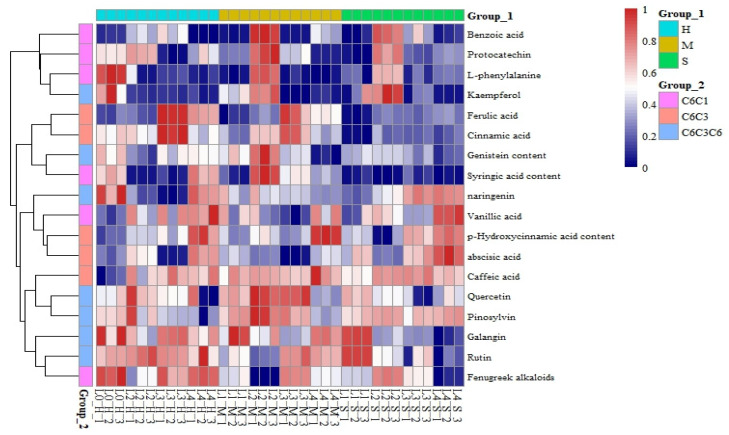
Cluster analysis of phenolic compounds in larch leaves under different light and moisture conditions. The color ranges from red to blue, indicating relative abundance from high to low (shown in the color scale on the right).

A total of 18 phenolic compounds were identified in this study of larch seedling leaves under coupled light–water treatments. In addition to the precursor compound L-phenylalanine, 18 phenolic compounds were identified. The structural types can be roughly divided into three categories: five phenolic acid compounds with C6-C1-type structural skeletons (benzoic acid, syringic acid, trigonelline, protocatechuic acid, and vanillic acid), five phenylpropanoid compounds with C6-C3-type structural skeletons (p-hydroxycinnamic acid, abscisic acid, ferulic acid, cinnamic acid, and caffeic acid), and seven C6-C3-C6 structural flavonoids (genistein, apigenin, naringenin, quercetin, kaempferol, galangin, rutin, and taxifolin). The content of the phenolic compounds in the leaves of larch seedlings significantly differed under the different treatment conditions (Figure 9). C6-C3-C6 phenols, including genistein and quercetin, accumulated more in L2 and L3. C6-C1 and C6-C3 phenols were mainly found in the L2 treatment, and C6-C1 phenols accumulated less under the L4 treatment.

## 4. Discussion

### 4.1. Effects of Light and Water on Plant Growth and Photosynthesis

Light and water are important environmental factors affecting plant growth, development, and reproduction [23]. Drought inhibits plant height, ground diameter, and biomass allocation, while shading reduces plant height and ground diameter [24]. When these two conditions coexist, moderate shading (with sufficient soil moisture) can alleviate the adverse effects of drought (with deep shading) on plant growth to a certain extent [25].

In this study, under certain soil water conditions, the biomass of branches, leaves, and roots decreased with decreasing light intensity. This result is consistent with the study of *Carpinus betulus* L. [26]. Some studies have shown that when plants are exposed to shade stress, they tend to have a decreasing base diameter and increasing plant height; that is, they show the characteristics of being “slender” [27]. In this study, the slender morphological characteristic appeared in larch seedlings in the 60% water supply treatment under 50% shading. We speculate that this may be an adaptation; by reducing the ground diameter, seedlings can use more photosynthetic products for vertical growth to obtain morelight. In this study, both drought and shading inhibited the growth of the basal diameter and plant height of larch seedlings. This is consistent with the research results of relevant scholars [28]. This may be because the net photosynthetic rate of the plant decreased under the influence of light and water conditions. The synthesis of photosynthetic products is affected, which limits the thickening and growth of plants.

Light and water affect photosynthesis by regulating photosynthetic indices such as Pn, Tr, and Gs [29]. In this study, Ci was positively correlated with Pn, Tr, and Gs, indicating that the decrease in photosynthesis was caused by stomatal factors hindering CO_2_ utilization [26]. Research has shown that the photosynthetic capacity of larch is greater under full light [30], and that moderate shading is beneficial for efficient photosynthesis [31]. This study showed that the photosynthetic capacity under L1-H and L2-M coupling was significantly higher than other treatments. We speculate that the level of photosynthesis is related to water supply under different light levels. Excessive shading will destroy the photosynthetic mechanism of larch seedlings and prevent them from growing normally [14]. In this study, we found that over-shading (90%), regardless of water supply, decreased photosynthetic capacity, which supported the above view.

### 4.2. Effects of Light and Water on Photosynthetic Pigments and Chlorophyll Fluorescence

Chlorophyll has the function of absorbing and transferring light photons and is an important component in the maintenance of the normal photosynthesis of plants. Its content is closely related to the site conditions of plant growth and the characteristics of plants [29]. *Chl a* and *Chl b* are the main photosynthetic pigments, and carotenoids can absorb light energy and transfer it to chlorophyll to assist plants in photosynthesis [32]. The content and proportion of these pigments are important indicators for plants to cope with environmental stress [33]. Studies have shown that chlorophyll content decreases with the increase in shading intensity [34]. In this study, we found that the changes in chlorophyll a and *Ch a+b* content under 80% water supply were consistent with the above view. This shows that the water supply is sufficient, and shading stress impaired their photosynthetic machinery. Previous studies on *Aloe vera* L. showed that the pigment content was the highest under 50% shade and 60% water supply. This result is consistent with the changes in the L2-M treatment group in this study. It shows that under moderate water supply conditions, the pigment content of leaves with light shading is greater than that of leaves with sufficient light [35].

Chlorophyll fluorescence, part of the photosynthetic process, mainly reflects the efficiency of light absorption, transmission, and utilization by plants and is commonly used to characterize the level of the photosynthetic efficiency of plants [36]. Fv/Fm is an indicator of the photoconversion efficiency of PSII. The higher the Fv/Fm, the higher the photoconversion efficiency of PSII [37]. Previous studies have found that the Fv/Fm of plants is generally 0.75~0.85. In this experiment, Fv/Fm was more than 0.75 under L2-M and L1-H treatments, there was no significant difference between the two treatment groups (*p* > 0.05), and the other treatment groups were lower than 0.75, indicating that the open proportion of the PSII reaction center in larch under the other coupling treatments decreased and the light energy utilization efficiency was reduced. Under the same soil water supply, Y(II) and qP in the severe shading (90%) treatment were significantly lower than those in the natural light and 50% shading treatments, while NPQ was opposite under H and M water conditions, indicating that excessive shading is not conducive to improving the utilization rate of light energy. This results in photoinhibition, high levels of nonphotochemical consumption of photosynthetic pigments, increased heat dissipation, and adverse conditions for plant leaves, thus reducing the photosynthetic efficiency of larch [26].

### 4.3. Effects of Light and Water on Total Phenols and Total Flavonoids

Plant secondary metabolites are products of the long-term evolution and have an environmental impact. They play an important role in improving plant self-protection, enhancing survival and competition ability, and coordinating relationships with the environment [38].

The results of existing studies on *Juglans sigillata* Dode showed that the total flavonoid content was highest under full light, and the total flavonoid content decreased significantly in more than 60% of the shaded plants [39]. The study of blueberry varieties showed that the flavonoid content was the highest when the light intensity was 50% [40]. In this study, we found that the above views were supported under different water supply. In the total flavonoid content of leaves, it was significantly accumulated under L1 light conditions under H treatment, and under M and S treatments, it was significantly accumulated under L2 light conditions. We speculate that the content of total flavonoids in leaves under different light treatments is related to water supply. Previous studies on total phenols of *Tetrastigma hemsleyanum* and total flavonoids of blueberry showed that heavy shading was not conducive to the accumulation of its content [40,41]. In this study, we found that under L4 light conditions, regardless of water supply, its content accumulation was significantly reduced. We speculated that the light intensity had a certain effect on the accumulation of total phenols and total flavonoids, and its content did not increase linearly with the increase in light intensity. However, there is an optimal light intensity and humidity [42]. Under this condition, the total phenol and total flavonoid content of larch plants was the highest. When the light intensity is greater than or less than this light intensity, the synthesis of photosynthetic products is inhibited, which further hinders the accumulation of total phenols and total flavonoids, thus affecting the metabolic ability to maintain the normal growth of plants [41].

### 4.4. Effects of Light and Water on Phenolic Compounds

Previous studies have found that C6-C3-C6 phenols, including genistein and quercetin, accumulate more under severe shading conditions, while C6-C1 and C6-C3 phenols accumulate mainly under mild shading conditions [43]. In this study, we found that C6-C3-C6 phenolic compounds accumulated more in L2 and L3 under the M water supply. C6-C1 and C6-C3 phenolic compounds accumulated more in L2 under M water supply, which had antibacterial and antioxidant capacity and improved the disease resistance of *larch* [44]. It indicated that larch had strong adaptability under this treatment, and it was speculated that the accumulation of phenolic substances under light intensity was related to water supply. However, previous studies on *larch* needles showed that the cinnamic acid content was the highest under 75% shading [45]. Therefore, the response mechanisms of phenolic substances to light intensity differ among species, and further metabolomics research is needed to determine the underlying mechanism involved. In summary, most of the phenolic compounds with protective effects accumulated in the L2 treatment, which may be indicative of the mechanisms by which mild shading (L2) promotes the adaptability of *larch*.

## 5. Conclusions

The adaptation of larch seedlings to light conditions depends on the supply of water. Under the condition of 40% soil saturated water content, regardless of the light conditions, water deficit is a limiting factor for the growth of larch seedlings. When 80% of the soil has a saturated water content, more light is conducive to seedling growth. When 60% of the soil has a saturated water content, a moderate shading environment is more favorable for seedling growth and the accumulation of metabolites. Taken together, these findings provide a theoretical basis for forest management and artificial strategies to promote the natural regeneration of *L. gmelinii,* which has a high economic value.

## Figures and Tables

**Table 1:** The coupling of light intensity and water conditions.

Light Conditions	Water Conditions	Mark
natural sunlight	80% soil saturated water content	L1-H
natural sunlight	60% soil saturated water content	L1-M
natural sunlight	40% soil saturated water content	L1-S
50% shading	80% soil saturated water content	L2-H
50% shading	60% soil saturated water content	L2-M
50% shading	40% soil saturated water content	L2-S
75% shading	80% soil saturated water content	L3-H
75% shading	60% soil saturated water content	L3-M
75% shading	40% soil saturated water content	L3-S
90% shading	80% soil saturated water content	L4-H
90% shading	60% soil saturated water content	L4-M
90% shading	40% soil saturated water content	L4-S

**Table 2:** Significance levels of light intensity, water deficit, and the effect of their interactions on the measured variables according to two-way ANOVA.

Variables	F-Values and Significant Levels		
	Water	Light	Water × Light
plant height increment	71.99 **	24.44 **	2.28 *
ground diameter increment	29.92 **	82.24 **	10.57 **
biomass—branch	0.10 NS	5.8 **	1.13 NS
biomass—root	0.9 NS	45.06 **	4.30 **
biomass—leaf	2.5 NS	18.40 **	5.50 **
R/S	1.2 NS	2.2 NS	2.64 *
Pn	413.35 **	42.03 **	10.36 **
Gs	46.95 **	20.2 **	5.47 **
Ci	13.21 **	82.95 **	5.74 **
Tr	1850.53 **	504.32 **	150.24 **
*Chl a*	78.14 **	47.42 **	10.27 **
*Chl b*	12.42 **	4.56 *	2.29 NS
*Car*	18.93 **	15.52 **	2.50 NS
*Chl a+b*	71.66 **	38.78 **	9.58 **
*Chl a/b*	0.35 NS	0.34 NS	1 NS
*Chl/Car*	2.33 NS	2.65 NS	1.03 NS
Fv/Fm	1.69 NS	23.88 **	3.67 **
qP	6.80 **	171.37 **	25.00 **
NPQ	2.49 NS	2.74 **	2.77 **
Y(II)	7.50 **	137.40 **	15.53 **
TP—leaf	36.02 **	13.6 **	0.886 NS
TP—branch	16.85 **	9.06 **	2.93 *
TP—root	28.04 **	2.38 NS	6.31 NS
TF—leaf	7.94 **	293.86 **	57.13 **
TF—branch	0.04 NS	22.15 **	16.74 **
TF—root	7.96 **	13.74 **	4.63 **

Note: The legend indicates the level of correlation. NS indicates no significant difference; * and ** indicate significant differences at *p* < 0.05 and *p* < 0.01, respectively.

## Data Availability

The original contributions presented in the study are included in the article, further inquiries can be directed to the corresponding authors.

## Abbreviations

R/S—root-shoot ratio; *Chl a*—chlorophyll a contents; *Chl b*—chlorophyll b contents; *Chl a+b*—chlorophyll a+b contents; *Chl a/b*—chlorophyll a/b; Car—carotenoid contents; Chl/Car—chlorophyll contents/carotenoid contents; F_V_/F_m_—maximum quantum efficiency of PSII photochemistry; qP—photochemical quenching; NPQ—nonphotochemical quenching; Y(II)—effective quantum yield of photosystem II; P_n_—net CO_2_ assimilation rate; Gs—stomatal conductance; Tr—transpiration rate; Ci—intercellular CO_2_ concentration; TP—total phenols; TF—total flavonoids.
